# Maternal Dietary Patterns and Fetal Growth: A Large Prospective Cohort Study in China

**DOI:** 10.3390/nu8050257

**Published:** 2016-04-28

**Authors:** Min-Shan Lu, Qiao-Zhu Chen, Jian-Rong He, Xue-Ling Wei, Jin-Hua Lu, Sheng-Hui Li, Xing-Xuan Wen, Fan-Fan Chan, Nian-Nian Chen, Lan Qiu, Wei-Bi Mai, Rui-Fang Zhang, Cui-Yue Hu, Hui-Min Xia, Xiu Qiu

**Affiliations:** 1 Division of Birth Cohort Study, Guangzhou Women and Children’s Medical Center, Guangzhou Medical University, 9 Junsui Road, Tianhe District, Guangzhou 510623, China; biglms@126.com (M.-S.L.); Hjr0703@163.com (J.-R.H.); xiaoshuierde@163.com (X.-L.W.); lujinhua10@gmail.com (J.-H.L); lishenghui1005@gmail.com (S.-H.L.); wen.xing.xuan@163.com (X.-X.W.); bigcsandy@hotmail.com (F.-F.C.); cnnfezx@163.com (N.-N.C.); mewmewprincess@hotmail.com (L.Q.); bighcy@163.com (C.-Y.H.); 2 Department of Obstetrics and Gynecology, Guangzhou Women and Children’s Medical Center, Guangzhou Medical University, 9 Junsui Road, Tianhe District, Guangzhou 510623, China; bigcqz@163.com (Q.-Z.C.); bigmwb@163.com (W.-B.M.); bigzsf@163.com (R.-F.Z.)

**Keywords:** dietary patterns, fetal growth, cluster analysis, prospective studies, China

## Abstract

There was limited evidence revealing the association of Chinese maternal dietary patterns with fetal growth. We aimed to examine the relationship of maternal dietary patterns during pregnancy to neonatal birth weight and birth weight for gestational age in a Chinese population. A total of 6954 mother-child pairs were included from the Born in Guangzhou Cohort Study. Maternal diet during pregnancy was assessed using a self-administered food frequency questionnaire. Cluster analysis was used to identify dietary patterns. The following six dietary patterns were identified: “Cereals, eggs, and Cantonese soups” (n 1026, 14.8%), “Dairy” (n 1020, 14.7%), “Fruits, nuts, and Cantonese desserts” (n 799, 11.5%), “Meats” (n 1066, 15.3%), “Vegetables” (n 1383, 19.9%), and “Varied” (n 1224, 17.6%). The mean neonatal birth weight Z scores of women in the above patterns were 0.02, 0.07, 0.20, 0.01, 0.06, and 0.14, respectively. Women in the “Fruits, nuts, and Cantonese desserts” and “Varied” groups had significantly heavier infants compared with those in the “Cereals, eggs, and Cantonese soups” group. Compared with women in the “Cereals, eggs, and Cantonese soups” group, those in the “Varied” group had marginally significantly lower odds of having a small-for-gestational age (SGA) infant after adjustment for other confounders (OR 0.77, 95% CI 0.57, 1.04, *p* = 0.08). These findings suggest that compared to a traditional Cantonese diet high in cereals, eggs, and Cantonese soups, a diet high in fruits, nuts, and Cantonese desserts might be associated with a higher birth weight, while a varied diet might be associated with a greater birth weight and also a decreased risk of having a SGA baby.

## 1. Introduction

Fetal growth is an important determinant of health and disease in a human’s entire lifetime. Studies suggest that restricted fetal growth is associated with an increased risk of childhood morbidity and chronic diseases in adulthood [1,2,3,4], while high birth weight has been associated with an increased risk of obesity [5], cancers [6,7,8], cardiovascular diseases, and diabetes [9].

Adequate maternal nutrition during pregnancy is vital for fetal growth [10]. As a modifiable factor, maternal diet receives considerable attention in previous studies of fetal growth. Most previous studies have been focused on assessing the association between single foods or nutrients and fetal growth and the results are inconsistent [11,12,13,14,15,16,17]. It is difficult to separate out the specific effects of single foods or nutrients on fetal growth because of the highly interrelated nature of dietary exposures [18]. Hence analysis assessing whole foods or dietary pattern analysis has been proposed as a useful method to address some of the problems in nutrient-based analyses and could be also more intuitive to public health diet recommendations [18]. 

Thus far, there are several studies that have investigated the relationships between maternal dietary patterns and fetal growth [19,20,21,22,23,24,25,26]. Most of the studies were conducted in Western countries, including the USA [19,25], Denmark [20], the UK [21], New Zealand [22], and Australia [26]. Only one study was conducted in an Eastern population (Japanese) [23]. There was limited evidence demonstrating the relationship between maternal dietary patterns and fetal growth in a Chinese population. Chinese pregnant women have complex and diverse eating habits, and follow a set of dietary customs that usually lack scientific justification [27,28]. Thus, further studies are necessary to elicit the role of maternal dietary patterns during pregnancy in fetal growth.

Hence, this study aims to examine the effect of different maternal dietary patterns during pregnancy on fetal growth in a Chinese population.

## 2. Materials and Methods

### 2.1. Study Design

The present study was based on the Born in Guangzhou Cohort Study (BIGCS), an ongoing population-based prospective cohort study from fetal life onwards that has been described in detail previously [29]. Briefly, BIGCS is conducted in the Guangzhou Women and Children’s Medical Center (GWCMC). From February 2012, pregnant women who lived in Guangzhou, attended their first routine antenatal examinations (normally around 16 weeks) at two campuses of the GWCMC, and intended to stay at Guangzhou with their child for at least three years, were considered eligible and were then invited to participate in the BIGCS. At the time of recruitment, participants were asked to fill in the comprehensive baseline questionnaires (Q1). At about 24 to 27 weeks they came back to GWCMC and finished the follow-up Q2 questionnaire. The protocols of the BIGCS were reviewed and approved by the Institutional Ethics Committee of the GWCMC. Written consent was obtained from all participants.

### 2.2. Population for Analysis

**Figure 1:**
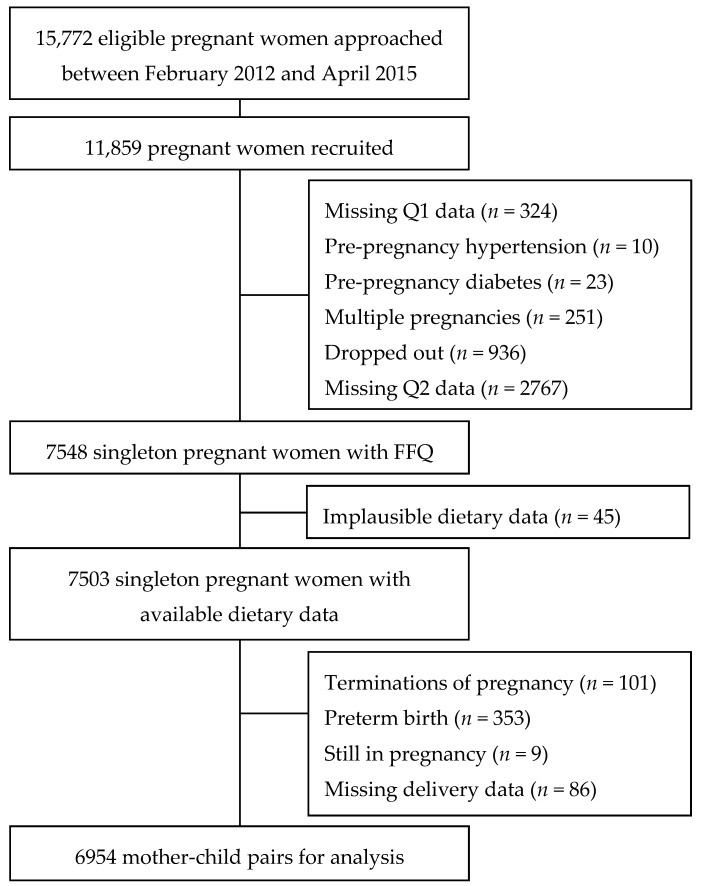
Selection process of study population in the Born in Guangzhou Cohort Study (BIGCS). FFQ refers to food frequency questionnaire.

For the present study, pregnant women, who were recruited between February 2012 and April 2015, and their infants were included as study subjects. A flowchart of the selection process of the study population is shown in Figure 1. Women who took part in the BIGCS tended to be slightly older and have a higher education than those who were eligible but did not participate (Table A1). 

### 2.3. Dietary Assessment

Information about dietary intake was obtained with a self-administered food frequency questionnaire (FFQ) at the Q2 interview. We evaluated the validity and reproducibility of this FFQ elsewhere, and the FFQ has been shown to be a valid and reliable instrument to assess dietary intake [29]. This FFQ consists of sixty-four specified food items as well as additional questions regarding cooking oil, beverages, and soup. For each food item, participants reported how often they had consumed it “in the past week”. Data on the amount consumed were not collected. Individual food items were combined into thirty food groups according to a similar nutrient profile or culinary uses. The frequencies of intake of food groups were calculated by summing the weekly consumption frequencies of grouped foods, assuming that different food items in the same group contributed equally. The percentages of weekly intake of the food groups were calculated as “frequency of the food group intake divided by total frequencies of food intake” for each participant.

### 2.4. Anthropometric Measurements at Birth

Information on birth weight and gestational age was obtained from the medical charts. Birth weight Z score was calculated on the basis of the birth weight reference in Guangzhou [30]. Small-for-gestational age (SGA) infants were defined as infants with birth weight below the 10th percentile of the Guangzhou gestational age- and sex-specific reference growth curves, while large-for-gestational age (LGA) infants were defined as infants with birth anthropometric measurements above the 90th percentile of the Guangzhou gestational age- and sex-specific reference growth curves [30].

### 2.5. Covariates

Several socio-demographic, health, and medical history characteristics were considered as possible confounders. Information regarding maternal age, education level (middle school or below, college, undergraduate, or postgraduate), monthly income (≤1500, 1501–4500, 4501–9000, or >9001 RMB), parity (0, ≥1), passive smoking during pregnancy (no, yes), alcohol drinking during pregnancy (no, yes), folic acid supplement use (no, started during first 10 weeks, or started pre- conception), pre-pregnancy maternal body measurements (weight, height) was obtained from the Q1 questionnaires. Pre-pregnancy body mass index (BMI) (kg/m^2^) was calculated by dividing weight in kilograms by height in meters squared. According to the Guidelines for Prevention and Control of Overweight and Obesity in Chinese Adults [31], study participants were divided into three groups as follows: BMI < 18.5 kg/m^2^ (underweight), BMI 18.5–23.9 kg/m^2^ (normal), BMI ≥ 24 kg/m^2^ (overweight or obese). The diagnosis of gestational diabetes mellitus (GDM) was made based on the outcomes of GDM screening [29].

### 2.6. Statistical Analyses

Percentages of weekly intake of the above thirty food groups were taken into account to construct dietary patterns. Cluster analysis was performed using the k-means procedure in the R version 3.2.3 (The R project, http://www.r-project.org). The K-means method was applied to classify participants into a predetermined number of mutually exclusive groups by comparing Euclidean distances between each participant and each cluster center in an interactive process until no further changes occur [32]. Several runs were conducted varying the number of clusters from two to six towards an optimal number. The final cluster solution was selected by comparing the ratio of between-cluster variance to within-cluster variance divided by the number of clusters. Based on the aforementioned determinations and on the nutritional meaningfulness of clusters, we selected the six-cluster solution as the most appropriate solution.

Descriptive statistics (*i.e.*, mean, standard deviation, frequencies, and percent frequencies) were reported for all socio-demographic factors (age, education level, monthly income), health characteristics (parity, passive smoking during pregnancy, alcohol drinking during pregnancy, pre-pregnancy BMI), and medical history characteristics (GDM). These variables were cross- tabulated by dietary patterns, and significant differences were assessed by ANOVA for continuous variables or chi-square tests for categorical variables.

Mean differences in birth weight Z score across the dietary patterns were assessed using general linear models. Multivariable linear regression was used to determine the association between different dietary patterns and birth weight Z score. Multivariable logistic regression was used to assess the association between the different dietary patterns and the risk of having a SGA or LGA baby. These models were adjusted for maternal age, education level, monthly income, pre-pregnancy BMI, parity, smoking during pregnancy, passive smoking during pregnancy, alcohol drinking during pregnancy, and folic acid supplement use. 

*p* < 0.05 was considered statistically significant for all statistical tests. All analyses were performed using R version 3.2.3 or SPSS software version 20.0 (SPSS, Inc., Chicago, IL, USA).

## 3. Results

### 3.1. Dietary Patterns

Six dietary patterns were identified (Table 1). We labeled them “Cereals, eggs, and Cantonese soups” (n 1026, 14.8%), “Dairy” (n 1020, 14.7%), “Fruits, nuts, and Cantonese desserts” (n 799, 11.5%), “Meats” (n 1066, 15.3%), “Vegetables” (n 1383, 19.9%), and “Varied” (n 1224, 17.6%), based on the food groups predominant in each cluster. “Cereals, eggs, and Cantonese soups”, representing a traditional Cantonese diet, had the highest content of staples such as rice, pasta, porridge, eggs, and Cantonese soups. “Dairy” had the highest content of dairy. “Fruits, nuts, and Cantonese desserts” had the highest content of fruits, nuts, and Cantonese desserts. “Meats” had the highest content of red meat and processed meat. “Vegetables” had the highest content of leafy and cruciferous vegetables. “Varied” was characterized by relatively high intakes of mixed food, including noodles, bread, root vegetables, melon vegetables, mushrooms, sea vegetables, bean vegetables, processed vegetables, poultry, animal organ meat, fish, other seafood, bean products, yoghourt, sweet beverages, puffed food, confectioneries, and snacks.

### 3.2. Study Population Characteristic

Table 2 shows subject characteristics across the six dietary patterns. There were significant differences in maternal age, education level, monthly income, parity, passive smoking during pregnancy, alcohol drinking during pregnancy, and GDM among subjects in these six groups. Women in the “Cereals, eggs, and Cantonese soups” group were younger and had lower educational level. Women in the “Dairy” group tended to be more nulliparous, have smaller monthly income, and were more likely to develop GDM. Women in the “Fruits, nuts, and Cantonese desserts” group tended to have higher monthly income and be drinkers. Women in the “Meats” group were more likely to be exposed to passive smoking. Women in the “Vegetables” group were older and tended to be multiparous. Women in the “Varied” group tended to have better education and less likely to drink alcohol and have passive smoking during pregnancy. No significant difference regarding folic acid supplement use and pre-pregnancy BMI was found among subjects in these six groups.

### 3.3. Modeling

Table 3 shows associations between the six dietary patterns and neonatal birth weight Z score. The mean neonatal birth weight Z scores of women in the “Cereals, eggs, and Cantonese soups”, “Dairy”, “Fruits, nuts, and Cantonese desserts”, “Meats”, “Vegetables”, and “Varied” groups, were 0.02, 0.07, 0.20, 0.01, 0.02, and 0.06, respectively. Compared with women in the “Cereals, eggs, and Cantonese soups” group, women in the “Fruits, nuts, and Cantonese desserts” and “Varied” groups had infants with significantly higher birth weight Z score (β 0.06, 95% CI 0.09, 0.26, *p* < 0.05 for “Fruits, nuts, and Cantonese desserts”, β 0.06, 95% CI 0.05, 0.20, *p* < 0.05 for “Varied”, respectively) in the crude model. The results were consistent with those in the adjusted models. None of the other dietary patterns were significant predictors of birth weight Z score neither in the crude nor adjusted models.

Table 4 shows associations between dietary patterns and birth weight for gestational age. Compared with women in the “Cereals, eggs, and Cantonese soups” group, those in the “Varied” group had significantly lower odds of having a SGA infant in the crude model (OR 0.69, 95% CI 0.51, 0.93, *p* < 0.05). The association was marginally significant after adjustment for socio-demographic and other confounders (OR 0.77, 95% CI 0.57, 1.04, *p* = 0.08). No significant difference in the risk of having a LGA infant was observed among subjects in these patterns neither in the crude nor adjusted models.

## 4. Discussion

In the present large prospective study, we investigated the association of maternal dietary patterns during pregnancy with neonatal birth weight Z score as well as the risk of having a SGA or LGA baby in a Chinese population. The dietary patterns examined emerged from the foods that this population normally consume, and cultural Cantonese cuisine was also captured. We found that, compared with women in the “Cereals, eggs, and Cantonese soups” group, those in the both “Fruits, nuts, and Cantonese desserts” and “Varied” groups had infants with higher birth weight Z scores. Women in the “Varied” group had a lower risk of having a SGA infant than those in the “Cereals, eggs, and Cantonese soups” group.

In our study, 14.8% of the women followed the “Cereals, eggs, and Cantonese soups” dietary pattern, characterized by a high intake of foods that are part of local eating habits, and representational of a typical traditional Cantonese diet. Women in the “Cereals, eggs, and Cantonese soups” group might be subjected to nutritional inadequacy, and a high intake of carbohydrate and low protein intake was associated with increased risk of low birth weight [33]. These factors could partly explain our findings that women in the “Cereals, eggs, and Cantonese soups” group had infants with relatively low birth weight. “Fruits, nuts, and Cantonese desserts” had the highest content of fruits, nuts and Cantonese desserts, and women who followed this dietary pattern had infants with higher birth weights than women in “Cereals, eggs, and Cantonese soups” (β 0.06, 95% CI 0.09, 0.26, *p* < 0.05). Women in “Varied” not only had infants with higher birth weights (β 0.06, 95% CI 0.05, 0.20, *p* < 0.05) but also had a marginally significant risk reduction of having a SGA infant (OR 0.77, 95% CI 0.57, 1.04, *p* = 0.08) in comparison to those in the “Cereals, eggs, and Cantonese soups” group. “Varied” was characterized by relatively high intakes of mixed food with high nutritional value, representing a more balanced and varied diet. Consumption of a variety of foods such as fruits, vegetables, and proteins is promoted in many dietary guidelines [34,35,36]. It is reasonable for us to conclude that women in the “Fruits, nuts, and Cantonese desserts” or “Varied” groups might have a higher intake of vitamins and minerals playing an essential role in fetal growth [37,38]. Previous studies have shown micronutrients contained in fruits and vegetables may also contribute to placental functions and optimal immune system functioning [39,40], which are important for fetal growth.

Maternal diet is critical to fetal growth [10]. Most of the previous studies of the relationship between dietary patterns in pregnancy and fetal growth were from western countries [19,20,21,22,24,25]. The pioneer study in Mexican-American mothers found that nutrient-dense (fruits, vegetables, and low-fat dairy) were associated with an increase in infant birth weight [19]. In addition, a second prospective study conducted in Denmark showed that those mothers who had a high intake of fruits, vegetables, fish, and poultry, had a significantly lower risk of having a SGA infant [20]. A third prospective study among Japanese women found that women in the “rice, fish, and vegetables” group during pregnancy might be associated with a large birth weight and a decreased risk of having a SGA infant [23]. Notwithstanding the variety of foods eaten across different countries and cultures and differences in the dietary patterns constructed, the present study and the previous studies mentioned above together suggest that heavier birth weights are associated with dietary patterns that contained vegetables and fruits. Besides the beneficial value of vegetables and fruits, our findings also emphasized the importance of the dietary variety. Dietary variety is one of the measures of dietary quality. A previous study in Spain found an inverse association between a high-quality diet and the risk of having a SGA baby [41]. Further studies are needed to detect the diet quality in our population.

Birth weight is an important health indicator and indirectly reflects the living conditions of populations. In our study, mothers in the “Cereals, eggs, and Cantonese soups” group were younger and had lower educational levels, while women in the “Fruits, nuts, and Cantonese desserts” and “Varied” groups had better socioeconomic status. In line with previous studies, our study also showed that better socioeconomic situations of mothers were commonly associated with healthy food consumption [22,31], as well as an appropriate birth weight [42]. As a result, for women who are younger and with lower socioeconomic status, a balanced and varied diet is more needed.

To our knowledge this is the first prospective study to examine the relationship between maternal dietary patterns and fetal growth in a Chinese population with a relatively large sample size. Compared with an analysis based on individual nutrients or food items, dietary pattern analysis is able to capture synergistic effects among various nutrients and types of food, and the findings from dietary pattern analysis might be more comprehensive and informative for clinical practice and public health. Rather than factor analysis, cluster analysis can provide a clear description of exactly what is being consumed, and focus more on groups of people with a good or poor nutritional status, which is more valuable for a nutrition intervention design to target pregnant women in need [43]. 

The present study had some limitations. Firstly, we did not collect data on portion sizes of food items, and were unable to calculate the amount of food consumption. As previous studies reported [29,44], frequencies of food intake were used as a proxy for a quantitative indicator in the present study. Thus, misclassification of exposure is inevitable. Such sources of error would bias results toward the null; hence, the strength of the observed associations might be underestimated. Secondly, we assessed food intake “in the past week”. The information during this short period might not be representative of dietary habits throughout pregnancy. However, previous studies have suggested that overall dietary patterns remain stable during pregnancy [45,46]. Thirdly, dietary consumption was based only on the frequency of food intake and information on portion size was not collected in the present study; thus, we were unable to adjust for total energy intake. Finally, residual confounding linked with both fetal growth and diet during pregnancy cannot be excluded, although we adjusted for several factors in the statistical analysis.

In conclusion, the present prospective study in Chinese pregnant women showed that compared to a traditional Cantonese diet high in cereals, eggs, and Cantonese soups, a diet high in fruits, nuts, and Cantonese desserts might be associated with a higher birth weight, while a varied diet might be associated with a greater birth weight and also a decreased risk of having a SGA baby. These findings provide scientific evidence to support the advice to have a balanced and varied diet for pregnant women in the Chinese population. Pregnant women are recommended to consume different kinds of food, and increase the proportion of vegetables and fruits in their diet, especially for those who are younger and have low socio-economic status. Further studies are needed to clarify the associations between dietary patterns and fetal growth in other Asian populations. 

## Figures and Tables

**Table 1:** Percentages (%) of weekly intake of 30 food groups assessed with an FFQ questionnaire across the six dietary patterns identified among 6954 pregnant Chinese women from the Born in Guangzhou Cohort Study.

Food group	Dietary Pattern											
	Cereals, Eggs, and Cantonese Soups		Dairy		Fruits, Nuts, and Cantonese Desserts		Meats		Vegetables		Varied	
	(*n* = 1026)		(*n* = 1020)		(*n* = 799)		(*n* = 1066)		(*n* = 1383)		(*n* = 1160)	
	Mean	SD	Mean	SD	Mean	SD	Mean	SD	Mean	SD	Mean	SD
Rice ^1^	16.4	3.9	10.9	3.4	10.2	3.4	12.7	3.2	12.0	3.5	8.2	2.8
Pasta ^1^	5.0	3.6	4.1	2.8	4.5	3.0	4.2	2.7	4.0	2.5	4.4	2.8
Noodles ^1^	2.2	2.6	1.6	2.0	2.5	2.6	2.0	2.2	2.0	2.1	2.8	3.0
Porridge ^1^	1.7	1.7	1.3	1.4	1.3	1.5	1.6	1.8	1.4	1.5	1.6	1.5
Bread ^1^	1.9	2.0	1.8	2.0	1.3	1.8	1.6	1.8	1.9	1.9	2.1	1.9
Leafy and cruciferous vegetables ^1^	7.9	2.9	10.6	3.3	9.2	3.5	10.5	3.7	17.9	3.8	10.1	3.0
Root vegetables ^1^	2.8	1.8	2.5	1.8	3.0	1.9	2.7	1.8	3.1	2.1	3.7	1.9
Melon vegetables ^1^	3.7	2.3	3.4	2.1	4.1	2.4	3.4	2.2	3.5	2.2	4.1	2.2
Mushrooms ^1^	1.1	1.1	1.0	1.2	1.2	1.1	1.0	1.0	1.1	1.1	1.5	1.2
Sea vegetables ^1^	0.6	0.9	0.6	0.8	0.8	0.9	0.6	0.9	0.7	0.9	1.0	1.0
Bean vegetables ^1^	1.3	1.3	1.2	1.1	1.3	1.1	1.3	1.1	1.3	1.1	1.6	1.2
Processed vegetables ^1^	0.5	0.9	0.3	0.7	0.5	1.0	0.4	0.8	0.4	0.9	0.6	1.0
Fruits ^1^	8.2	2.8	8.1	3.0	15.4	3.9	7.5	2.9	7.7	2.9	7.7	2.4
Red meat ^1^	6.8	2.7	7.8	3.1	7.0	3.0	14.4	3.4	7.6	3.0	7.2	2.5
Poultry ^1^	2.5	1.8	2.4	1.7	2.0	1.6	2.4	2.0	2.4	1.8	2.6	1.9
Animal organ meat ^1^	0.9	1.3	0.9	1.4	0.8	1.1	1.0	1.4	0.9	1.2	1.2	1.5
Processed meat ^1^	0.3	0.8	0.3	0.9	0.3	0.7	0.4	0.9	0.2	0.7	0.3	0.7
Eggs ^1^	5.9	2.9	5.8	2.5	5.4	2.5	5.5	2.8	5.1	2.3	5.4	2.3
Fish ^1^	3.0	2.0	3.0	2.0	2.6	1.8	2.9	2.1	2.9	1.8	3.1	1.9
Other seafood ^1^	1.0	1.2	0.9	1.1	1.0	1.2	0.9	1.1	0.9	1.1	1.2	1.3
Bean products ^1^	4.1	2.8	3.3	2.4	4.5	2.8	3.7	2.5	3.8	2.5	6.8	3.6
Nuts ^1^	3.8	2.7	4.1	2.9	4.5	2.9	3.3	2.5	3.6	2.5	4.3	2.4
Dairy ^1^	5.9	3.1	13.1	4.1	5.8	3.2	5.3	2.9	5.1	2.9	5.1	2.7
Yoghourt ^1^	2.0	2.3	1.4	1.8	2.1	2.3	1.7	2.0	1.8	2.1	2.4	2.2
Sweet beverages ^1^	1.6	2.6	1.2	2.2	1.4	2.2	1.2	1.9	1.2	2.0	1.9	3.1
Cantonese desserts ^1^	0.2	0.7	0.3	1.0	0.4	1.1	0.2	0.8	0.2	0.7	0.3	0.8
Cantonese soups ^1^	3.9	2.5	3.6	2.3	3.0	2.2	3.7	2.3	3.2	2.2	3.1	2.1
Puffed food ^1^	0.2	0.6	0.2	0.5	0.2	0.5	0.2	0.5	0.2	0.5	0.3	0.7
Confectioneries ^1^	1.6	2.1	1.3	2.0	1.7	2.2	1.3	1.8	1.4	2.0	2.2	2.5
Snack ^1^	2.9	2.8	2.8	2.6	2.1	2.2	2.3	2.3	2.6	2.3	3.2	2.4

^1^ Percentage values (%), calculated as frequency of the food group intake divided by total frequencies of food intake. The highest mean values are underlined.

**Table 2:** Characteristics of the participants across the six dietary patterns identified by cluster analysis among 6954 Chinese pregnant women from the Born in Guangzhou Cohort Study. GDM refers to gestational diabetes mellitus.

Characteristics	Dietary Pattern						*p* Value *
	Pattern ^1^	Dairy	Pattern ^2^	Meats	Vegetables	Varied	
	(*n* = 1026)	(*n* = 1020)	(*n* = 799)	(*n* = 1066)	(*n* = 1383)	(*n* = 1160)	
Age, years, mean ± SD	28.8 ± 3.3	28.9 ± 3.5	28.9 ± 3.1	29.0 ± 3.3	29.2 ± 3.4	29.1 ± 3.3	0.001
Education level, n (%)							<0.001
Middle school or below	97 (9.5)	99 (9.7)	75 (9.4)	73 (6.8)	127 (9.2)	101 (6.1)	
College	287 (28.0)	269 (26.4)	173 (21.7)	277 (26)	356 (25.7)	320 (19.3)	
Undergraduate	552 (53.8)	546 (53.5)	436 (54.6)	601 (56.4)	753 (54.4)	949 (57.2)	
Postgraduate or above	90 (8.8)	106 (10.4)	115 (14.4)	115 (10.8)	147 (10.6)	290 (17.5)	
Monthly income, Yuan, n (%)							<0.001
≤1500	86 (8.4)	99 (9.7)	86 (10.8)	96 (9.0)	137 (9.9)	148 (8.9)	
1501–4500	370 (36.1)	383 (37.5)	197 (24.7)	366 (34.3)	437 (31.6)	405 (24.4)	
4501–9000	421 (41.0)	380 (37.3)	359 (44.9)	425 (39.9)	575 (41.6)	722 (43.5)	
>9001	135 (13.2)	130 (12.7)	135 (16.9)	156 (14.6)	204 (14.8)	344 (20.7)	
Refused to answer	14 (1.4)	28 (2.7)	22 (2.8)	23 (2.2)	30 (2.2)	41 (2.5)	
Parity, n (%)							<0.001
Nulliparous	922 (89.9)	939 (92.1)	728 (91.1)	907 (85.1)	1155 (83.5)	1433 (86.3)	
Multiparous	104 (10.1)	81 (7.9)	71 (8.9)	159 (14.9)	228 (16.5)	227 (13.7)	
Passive smoking during pregnancy, n (%)	337 (32.8)	311 (30.5)	231 (28.9)	368 (34.5)	406 (29.4)	454 (27.3)	0.001
Alcohol drinking during pregnancy, n (%)	236 (23.0)	308 (30.2)	298 (37.3)	285 (26.7)	192 (13.9)	292 (17.6)	<0.001
Folic acid supplement use, n (%)							0.109
No	91 (8.9)	89 (8.7)	60 (7.5)	99 (9.3)	116 (8.4)	121 (7.3)	
Started during first 10 weeks	514 (50.1)	474 (46.5)	352 (44.1)	500 (46.9)	667 (48.2)	773 (46.6)	
Started pre- conception	421 (41.0)	457 (44.8)	387 (48.4)	467 (43.8)	600 (43.4)	766 (46.1)	
Pre-pregnancy BMI, kg/m^2^, n (%)							0.113
<18.5	238 (23.2)	266 (26.1)	192 (24.0)	239 (22.4)	326 (23.6)	373 (22.5)	
18.5–23.9	695 (67.7)	635 (62.3)	535 (67.0)	694 (65.1)	895 (64.7)	1109 (66.8)	
≥24	80 (7.8)	102 (10.0)	68 (8.5)	114 (10.7)	145 (10.5)	153 (9.2)	
Missing	13 (1.3)	17 (1.7)	4 (0.5)	19 (1.8)	17 (1.2)	25 (1.5)	
GDM, n (%)	101 (10.0)	126 (12.7)	78 (10.1)	121 (11.6)	119 (8.8)	173 (10.6)	0.048

* ANOVA and Chi square tests were used to test differences between the patterns. ^1^ “Cereals, eggs, and Cantonese soups”. ^2^ “Fruits, nuts, and Cantonese desserts”.

**Table 3:** Association between dietary patterns and neonatal birth weight Z score.

Birth Weight z Score	Dietary Pattern					
	Cereals, Eggs, and Cantonese Soups	Dairy	Fruits, Nuts, and Cantonese Desserts	Meats	Vegetables	Varied
	(*n* = 1026)	(*n* = 1020)	(*n* = 799)	(*n* = 1066)	(*n* = 1383)	(*n* = 1160)
Mean (95% CI)	0.02 (−0.04, 0.08) ^a,b^	0.07 (0.01, 0.13) ^c^	0.20 (0.13,0.26) ^a,c,d,e^	0.01 (−0.05, 0.07) ^d,f^	0.06 (0.01, 0.11) ^e^	0.14 (0.10, 0.19) ^b,f^
Crude β (95% CI)	Reference	0.02 (−0.04, 0.13)	0.06 (0.09, 0.26) *	0.01 (−0.09, 0.07)	0.02 (−0.04, 0.12)	0.06 (0.05, 0.20) *
Adjusted β (95% CI) ^1^	Reference	0.02 (−0.04, 0.13)	0.05 (0.07, 0.24) *	-0.01 (−0.10, 0.06)	0.01 (−0.04, 0.11)	0.04 (0.02, 0.17) *
Adjusted β (95% CI) ^2^	Reference	0.02 (−0.04, 0.13)	0.06 (0.08, 0.26) *	0.00 (−0.09, 0.07)	0.02 (−0.03, 0.12)	0.06 (0.05, 0.20) *
Adjusted β (95% CI) ^3^	Reference	0.02 (−0.04, 0.13)	0.06 (0.09, 0.27) *	-0.01 (−0.10, 0.07)	0.02 (−0.04, 0.12)	0.05 (0.05, 0.19) *
Adjusted β (95% CI) ^4^	Reference	0.02 (−0.03, 0.13)	0.05 (0.07, 0.24) *	-0.01 (−0.11, 0.05)	0.01 (−0.04, 0.11)	0.04 (0.01, 0.16) *

^a, b, c, d, e^^, f^ Mean values within a row with unlike superscript letters were significantly different (*p* < 0·05, Tukey–Kramer’s adjustment for Mixed comparisons in general linear models). * *p* < 0.05. ^1^ Adjusted for maternal age, education level, and monthly income. ^2^ Adjusted for parity, passive smoking during pregnancy, and alcohol drinking during pregnancy. ^3^ Adjusted for GDM. ^4^ Adjusted for maternal age, education level, monthly income, parity, passive smoking during pregnancy, alcohol drinking during pregnancy, folic acid supplement use, pre-pregnancy BMI, and GDM.

**Table 4:** Odds ratios (ORs) and 95% confidence intervals (95% CIs) of birth weight for gestational age to six dietary patterns identified among 6954 pregnant women from the Born in Guangzhou Cohort Study. SGA refers to small-for-gestational age; LGA refers to large-for-gestational age.

Birth Weight for Gestational Age	Dietary Pattern					
	Cereals, Eggs, and Cantonese Soups	Dairy	Fruits, Nuts, and Cantonese Desserts	Meats	Vegetables	Varied
	(*n* = 1026)	(*n* = 1020)	(*n* = 799)	(*n* = 1066)	(*n* = 1383)	(*n* = 1160)
SGA (n, %)	89 (8.7)	80 (7.8)	54 (6.8)	86 (8.1)	94 (6.8)	102 (6.1)
Crude OR (95% CI)	1.00 (Reference)	0.90 (0.65-1.23)	0.76 (0.54-1.08)	0.92 (0.68-1.26)	0.77 (0.57-1.04)	0.69 (0.51-0.93) *
Adjusted OR (95% CI) ^1^	1.00 (Reference)	0.89 (0.65-1.22)	0.80 (0.56-1.14)	0.94 (0.69-1.28)	0.78 (0.58-1.06)	0.75 (0.56-1.01)
Adjusted OR (95% CI) ^2^	1.00 (Reference)	0.90 (0.66-1.23)	0.77 (0.54-1.09)	0.92 (0.68-1.26)	0.77 (0.57-1.04)	0.69 (0.51-0.93) *
Adjusted OR (95% CI) ^3^	1.00 (Reference)	0.89 (0.65-1.23)	0.72 (0.50-1.03)	0.94 (0.69-1.29)	0.76 (0.56-1.04)	0.70 (0.52-0.95) *
Adjusted OR (95% CI) ^4^	1.00 (Reference)	0.87 (0.63-1.21)	0.76 (0.53-1.10)	0.95 (0.69-1.30)	0.77 (0.56-1.05)	0.77 (0.57-1.04)
LGA (n, %)	103 (10.0)	106 (10.4)	96 (12.0)	87 (8.2)	147 (10.6)	194 (11.7)
Crude OR (95% CI)	1.00 (Reference)	1.04 (0.78-1.38)	1.22 (0.91-1.64)	0.80 (0.59-1.07)	1.07 (0.82-1.39)	1.19 (0.92-1.53)
Adjusted OR (95% CI) ^1^	1.00 (Reference)	1.03 (0.78-1.38)	1.17 (0.87-1.57)	0.78 (0.58-1.06)	1.04 (0.80-1.36)	1.11 (0.86-1.43)
Adjusted OR (95% CI) ^2^	1.00 (Reference)	1.03 (0.77-1.37)	1.21 (0.90-1.62)	0.79 (0.59-1.07)	1.08 (0.82-1.41)	1.19 (0.93-1.54)
Adjusted OR (95% CI) ^3^	1.00 (Reference)	1.04 (0.77-1.36)	1.22 (0.90-1.64)	0.77 (0.57-1.04)	1.06 (0.81-1.38)	1.17 (0.91-1.51)
Adjusted OR (95% CI) ^4^	1.00 (Reference)	1.01 (0.75-1.35)	1.14 (0.84-1.54)	0.75 (0.56-1.02)	1.03 (0.79-1.36)	1.10 (0.85-1.42)

* *p* < 0.05. ^1^ Adjusted for maternal age, education level, and monthly income. ^2^ Adjusted for parity, passive smoking during pregnancy, and alcohol drinking during pregnancy. ^3^ Adjusted for GDM. ^4^ Adjusted for maternal age, education level, monthly income, parity, passive smoking during pregnancy, alcohol drinking during pregnancy, folic acid supplement use, pre-pregnancy BMI, and GDM.

## Abbreviations

The following abbreviations are used in this manuscript:

FFQ	Food frequency questionnaire
SGA	Small- for-gestational age
LGA	Large- for-gestational age

## Appendix

**Table A1:** Comparison of characteristics among women who agreed and disagreed to participate in BIGCS.

Characteristics	Agreed	Disagreed	*p* Value *
N	11859	3806	
Age (years), mean (SD)	29.0 (3.4)	28.7 (3.5)	<0.001
Education level, n (%)			
High school or below	1303 (11.0)	616 (16.2)	<0.001
College	3029 (25.5)	1063 (27.9)	
Undergraduate	6157 (51.9)	1718 (45.1)	
Postgraduate	1370 (11.6)	409 (10.8)	

* Significant differences across groups were tested by using t test or chi-square test.
